# CADAVERIC STUDY ON THE LEARNING CURVE OF THE TWO-APPROACH GANZ PERIACETABULAR OSTEOTOMY

**DOI:** 10.1590/1413-785220162402142388

**Published:** 2016

**Authors:** Fernando Portilho Ferro, Leandro Ejnisman, Helder Souza Miyahara, Christiano Augusto de Castro Trindade, Antônio Faga, José Ricardo Negreiros Vicente

**Affiliations:** 1. Universidade de São Paulo, Faculdade de Medicina, Hospital das Clínicas, Institute of Orthopedics and Traumatology, São Paulo, SP, Brazil

**Keywords:** Osteotomy, Hip dislocation, Congenital, Arthritis, Autopsy, Cadaver

## Abstract

**Objective:**

: The Bernese periacetabular osteotomy (PAO) is a widely used technique for the treatment of non-arthritic, dysplastic, painful hips. It is considered a highly complex procedure with a steep learning curve. In an attempt to minimize complications, a double anterior-posterior approach has been described. We report on our experience while performing this technique on cadaveric hips followed by meticulous dissection to verify possible complications.

**Methods:**

: We operated on 15 fresh cadaveric hips using a combined posterior Kocher-Langenbeck and an anterior Smith-Petersen approach, without fluoroscopic control. The PAO cuts were performed and the acetabular fragment was mobilized. A meticulous dissection was carried out to verify the precision of the cuts.

**Results:**

: Complications were observed in seven specimens (46%). They included a posterior column fracture, and posterior and anterior articular fractures. The incidence of complications decreased over time, from 60% in the first five procedures to 20% in the last five procedures.

**Conclusions:**

: We concluded that PAO using a combined anterior-posterior approach is a reproducible technique that allows all cuts to be done under direct visualization. The steep learning curve described in the classic single incision approach was also observed when using two approaches. Evidence Level: IV, Cadaveric Study.

## INTRODUCTION

In 1988, Ganz et al.^1^ first described the Bernese periacetabular osteotomy (PAO) technique. Today, it is widely recognized as an effective technique for the treatment of painful, non-arthritic, dysplastic hips.^2^ In this patient group, early symptoms are caused by structural instability and acetabular rim overload, sometimes with labral hypertrophy and tearing. These changes usually cause pain before arthritic changes ensue. After articular cartilage damage, joint space narrowing and osteophyte formation (i.e. arthritis), the benefits of this procedure tend to wane.^1^^,^ 2

During a PAO, a sequence of cuts are performed around the acetabulum, which allows it to be re-oriented to improve coverage of the femoral head. The original description of this technique utilized a single anterior approach.^1^ When such an approach is chosen, the posterior cuts (ischial cut and retroacetabular cut) are performed with an incomplete view, relying on the surgeon's expertise to verify the correct orientation of the osteotome, which may result in greater risk for iatrogenic neurovascular injury and/or fracture. Later, surgeons advocated the use of fluoroscopic imaging to facilitate the osteotomy. 

Studies have demonstrated this procedure has a steep learning curve.^3^^,^ 4 To minimize approach-related complications, different approach variations have been described, such as the modified Smith-Petersen, ilioinguinal and direct anterior approaches.^5^ Although these approaches are somewhat different, all anterior approaches share the disadvantage of providing limited exposure for the ischial osteotomy. While realizing this difficulty and in an attempt to improve exposure for the ischial cut, different authors have described variations of the technique using a secondary posterior, medial thigh and suprapubic incisions.^6^^-^ 9

A dual anterior-posterior approach technique has been proposed to improve visualization and osteotomy precision. A posterior Kocher-Langenbeck approach is employed to expose the posterior aspect of the acetabulum and ensure the ischial and retroacetabular cuts are performed precisely, under direct vision and with less use of fluoroscopy.^6^ A recent study compared the outcomes of PAO's performed using a single approach and this dual anterior-posterior approach. The authors reported similar operative times and improvement of Wiberg's and Sharp's angles in both groups.^6^

From an anterior approach alone, it is impossible to assess the safety of posterior neurovascular structures around the ischium, which are close to the osteotome and therefore at potential risk.^10^ Also, C-arm guidance is the only method the surgeon has to avoid medial or lateral deviation of the osteotome. If excessive lateral deviation of the osteotome occurs, the sciatic nerve is at risk and damage to this nerve has been previously reported.^11^

In this study, we performed the periacetabular osteotomy on cadaveric specimens using the anterior-posterior dual approach technique. After completing the cuts, we performed a meticulous dissection of the hip joint. We hypothesized that complications would be more common during the first surgeries, and then gradually become less frequent as the team's experience improved. We also anticipated that the dissection of cadaveric specimens after the osteotomies would allow for a thorough assessment of complications.

## METHODS

We utilized 15 fresh cadaveric specimens. We excluded specimens with incision scars around the hip joint and any known history of hip pathology, fracture or surgery. The study was approved by the morgue administration service in our institution by protocol number 296/12. 

The procedure started in a lateral decubitus, using the posterior Kocher-Langenbeck approach.^12^ The incision began 6cm inferior to the posterior superior iliac spine (PSIS) and continued distally along the fibers of the gluteus maximus up to the level of the greater trochanter.

**Figure 1.**
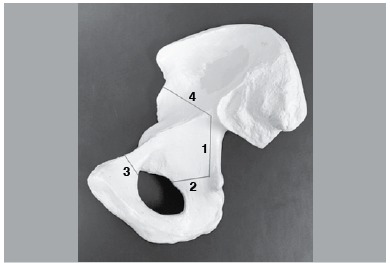
Bernese Periacetabular osteotomy cuts. Inner pelvis view. 1) Retroacetabular cut; 2) Ischial cut; 3) Superior pubic ramus cut; 4) Iliac wing cut.

The gluteus maximus was bluntly opened along the direction of its fibers for exposure of the short external rotators. The sciatic nerve was identified and protected. The piriformis tendon was released from its femoral insertion. The obturator internus and gemelli were released, to fully expose the posterior aspect of the lesser sciatic notch. The quadratus femoris was not released from the femur to protect the medial circumflex artery. Through subperiosteal dissection, we could visualize the posterior acetabular column and ischium. The retroacetabular osteotomy was then performed with a combination of different osteotome sizes. This osteotomy began 1cm above the ischial spine and continued proximally, always keeping a safe distance of 1cm from the margin of the posterior column and being parallel to it. The margin of the posterior column could be palpated at all times to verify the correct direction of the osteotomy. (Figure 1) 

The second osteotomy was the ischial osteotomy. It started at the inferior margin of the retroacetabular osteotomy along the infracotyloid groove. It was done at an angle of 120º to the retroacetabular osteotomy. This cut was done under direct vision and with continuous protection of the sciatic nerve. The cut was directed anteriorly and was completed just inferior to the acetabulum anteriorly.

After completing these two first osteotomies, the cadaver was turned to dorsal decubitus. A modified ilioinguinal approach was performed. Incision started 6cm above the anterior superior iliac spine (ASIS) and continued distally along the iliac crest. The lateral femoral cutaneous nerve was identified and retracted. Because we had already performed two of the osteotomies from the posterior approach, the anterior dissection could be kept smaller, just the necessary for exposure of the superior pubic ramus and iliac wing. The hip was flexed to relax the iliopsoas muscle, and a subperiosteal dissection was carried out along the superior pubic ramus. Hip flexion was of utmost importance to reduce muscle tension and allow enough exposure of the superior pubic ramus. If visualization was not adequate, the iliacus muscle was further released from the inner pelvic wall.

A retractor was positioned medial to the iliopectineal eminence, and the third osteotomy was performed at the pubic ramus, 1cm medial to the iliopectineal eminence.

Then attention was turned to the iliac wing. The iliacus muscle was detached from the inner pelvic wall by periosteal dissection. The proximal end of the retroacetabular osteotomy was palpable from inside the pelvis. The final (iliac) osteotomy began just below the ASIS and extended vertically, along the inner pelvic wall and finishing 1cm short of the margin of the posterior column, where it connected with the retroacetabular cut that was previously done from the posterior approach.

The completion of all osteotomies was verified by moving the acetabular fragment with an osteotome placed in the osteotomy gap. A 5mm Schanz screw was placed in the iliac crest to be used as a joystick and the fragment was mobilized.

After verification that the fragment was completely mobile, attention was turned to verify the adequacy of the osteotomies. From the anterior approach, the incision was extended distally. A Smith-Petersen approach was done to expose the anterior capsule. The capsule was incised longitudinally, and an anterior capsulectomy was performed. This allowed an anterior dislocation without tension, to avoid any secondary fractures due to forceful manipulation. The articular surface of both the acetabulum and femoral head was then inspected and palpated. Any intra-articular extensions were identified, and an attempt was made to verify which of the osteotomies was implicated. In addition, we extended the dissection medially to verify any evidence of damage to the obturator bundle due to its proximity to the pubic ramus osteotomy.

**Figure 2.**
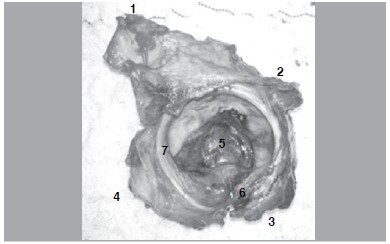
Acetabular fragment removed after osteotomy, external view. After soft tissue release, the fragment can be completely inspected. 1) Iliac crest and iliac osteotomy; 2) Retroacetabular osteotomy; 3) Ischial cut; 4) Iliopectineal eminence and pubic cut; 5) Cotyloid fossa; 6) Transverse ligament; 7) Labrum.

From the posterior approach, further dissection for capsule exposure and a posterior capsulectomy was also done. The joint was then dislocated, without soft tissue tension. The articular surface was again inspected, as done from the anterior approach. Additionally, the posterior approach allowed for a meticulous inspection of the posterior column after elevating the abductor muscles. We verified it thoroughly for any violation of its continuity, from the ischial tuberosity distally to the sacroiliac joint proximally. (Figure 2) At this point, we also verified any evidence of neurovascular damage, specifically to the sciatic nerve and pudendal bundle, as well as other structures emerging from the greater sciatic notch. 

### Data Analysis

Data analysis was done using a commercially available software (Excel 2013, Microsoft, Redmond, WA). All data displayed - if not otherwise stated - is expressed in means; the minimal and maximal values are reported as range. The confidence interval value used was 95%.

## RESULTS

All procedures were performed from July 2012 to December 2012. Specimen's demographics are summarized in Table 1. The incidence and type of complications are summarized in Table 2.

**Table 1.** Specimens' demographics.

	**Average**	**Range**	**95% Confid. Interval**
Age	65.2 years	33 - 80	61.2 - 68.7
Height	168 cm	148 - 177	165 - 170
Weight	63.6 kg	38 - 86	58.8 - 68.4
BMI (kg/m2)	22.5	12.4 - 31.9	20.8 - 24.2
Gender	6 females / 9 males		

**Table 2.** Complications observed after extensive dissection following the PAO.

**Specimen number**	**Complication type**
1	-
2	Anterior column fracture
3	Low transverse articular fracture
4	-
5	High transverse articular fracture
6	Posterior column fracture at manipulation
7	-
8	Low transverse articular fracture
9	-
10	-
11	Low transverse articular fracture
12	-
13	-
14	-
15	-

**Figure 3.**
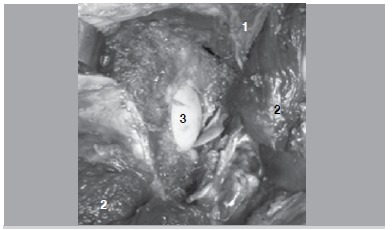
Femoral head exposure after articular extension of the retroacetabular cut. Image was obtained after thorough cadaveric dissection. 1) Iliac wing; 2) Reflected musculature; 3) Exposed femoral head cartilage.

In six cases (40%), we observed some form of complication: in one case, an anterior column fracture was observed. In another case, the posterior column was fractured during manipulation. In four cases, a transverse intra-articular extension was observed after dissection and surgical hip dislocation. (Figure 3) In two cases, these fractures were non-displaced and only visible after meticulous dissection and manipulation.

During dissection, we did not observe any macroscopic evidence of vascular or neurologic injury.

The rate of complications had a "learning curve" effect. During the first five procedures, a complication rate of 60% was observed. In the last five procedures, the complication rate dropped to 20%.

## DISCUSSION

To our knowledge, this is the first cadaveric study to evaluate the learning curve and potential complications of the periacetabular osteotomy using a dual anterior-posterior technique. This dual approach technique was first described by Kim et al.^6^ The secondary incision is posterior, using the Kocher-Langenbeck approach. The major advantage is the absence of intrapelvic dissection and manipulation, which potentially protects inner neurovascular structures. The dual anterior-posterior approach allows direct visualization of all bone cuts, potentially decreasing the risk of iatrogenic fractures and violation of the posterior column. In their study, Kim et al. described their experience while comparing the single and double approach techniques. They found no difference in patient outcomes, operation time and complication rates.^6^

We observed an anterior column intra-articular fracture as an extension of the superior pubic ramus osteotomy. In this specimen, a tight iliopsoas muscle impaired exposure of the correct osteotomy site. The osteotomy was done too close to the iliopectineal eminence, which probably lead to the fracture during manipulation. In another case, we observed a posterior column violation during manipulation of the acetabular fragment. That shows that even if the osteotomies are done precisely, such a complication may still occur due to forceful manipulation. This highlights the fact that the acetabular reorientation should never be attempted before verifying that all osteotomies are adequate and that the acetabular fragment is free. We also observed low and high transverse articular fractures. In both cases, this fracture was an extension of the retroacetabular cut. This is probably the most difficult osteotomy on the whole procedure. While doing it, it is of paramount importance to keep a safe 1cm distance from the border of the posterior column so as not to break it. However, keeping too large of a distance from the posterior column means being too close to the joint, and that is exactly what happened in these transverse fracture cases. 

The PAO is an extensively studied technique that provides pain relief and prevention of osteoarthritis (OA) in patients with hip dysplasia.^13^ The best indication would be a young patient with hip dysplasia, with abnormal Wiberg and Sharp angles. This patient typically has a normal range of motion and normal muscular function. Radiographs show decreased femoral head coverage, while joint space is normal. MRI's show a hypertrophic labrum and sometimes signs of overload to the acetabular rim subchondral bone (bone edema). Ideally, this patient will not have full thickness chondral lesions.^13^

**Figure 4.**
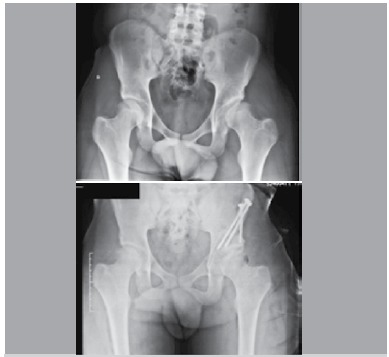
Case example: comparison of pre-operative and post-operative radiographs after a PAO done with a dual approach technique. The center-edge angle increased from 7 to 22 degrees.

Using the PAO technique, it is possible to change the center-edge angle by changing the acetabular orientation. (Figure 4) Consequently, body weight is evenly distributed across a larger portion of the joint cartilage, reducing acetabular rim overload. Most centers advise against doing this procedure on older patients with joint space narrowing, since the risk of persistent pain would not justify such a large intervention. For these patients, total hip replacement should be considered.^1^^,^ 2 ^,^ 13

The abundance of contact between bony surfaces allows for predictable bone healing. Also, it does not compromise the pelvic morphology^14^ and does not alter the results of a future total hip replacement for patients who develop arthritis after the osteotomy.^15^ A recent study has demonstrated that this technique is cost-effective, which highlights its relevance in preventing or delaying the onset of hip arthritis in dysplastic patients, thus reducing future costs related to total hip replacements.^16^

Despite its increased application and popularity, the PAO remains a highly complex procedure. Major reported complications include avascular necrosis, neurological dysfunction (femoral, sciatic and fibular), major blood loss, posterior column discontinuity, intra-articular fractures, deep vein thrombosis, undercorrection and also overcorrection.^1^^,^ 17 ^,^ 18 Although most centers describe the use of a classic single incision technique, different independent groups have published their experience on using two incisions to perform a PAO. They have modified the technique by employing a posterior, medial or suprapubic secondary incision, in an attempt to optimize visualization and minimize approach-related complications.^6^^-^ 9 The improved visualization obtained by using two approaches could possibly decrease complication rates in centers where this procedure is not done frequently.

The greatest benefit of performing this procedure in cadavers is the possibility of doing an extensive dissection after the cuts were completed and the acetabular fragment was mobilized. During such dissection, it was possible to obtain a more consistent understanding of this region's intricate anatomy, which may potentially help prevent the occurrence of complications when performing it in live patients. Such an extensive diagnostic dissection would be not ethically feasible in a live patient, due to the extensive soft tissue damage. 

A steep learning curve has been reported for the PAO.^1^^-^ 3 ^,^ 19 It has been proposed that most complications tend to occur in the first 20-30 cases done by a given surgeon.^1^^,^ 4 ^,^ 17 ^,^ 20 Therefore, prior surgery training on cadaveric specimens is strongly recommended.^1^ Several studies have recognized the PAO's associated risk of neurovascular injury, blood loss and intra-articular fracture associated with doing the procedure without complete exposure.^7^^,^ 10 Through cadaveric training, surgeons have the opportunity to become familiar with the intricate tridimensional nature of the osteotomy, while learning how minimal deviations from the correct technique may compromise the procedure's success. We observed a clear drop in complication incidence as we progressed. In the first five specimens, there was a 60% complication rate. This rate dropped to 20% in the last five cases. After a detailed dissection of all operated cases, we could observe which of the osteotomies was at an incorrect position, allowing us to adapt our technique accordingly for the following cases. 

Finally, we must advise that two of the intra-articular extensions were initially non-displaced and only visible after meticulous dissection. Based on this finding, we recommend that surgeons scrutinize post-operative radiographs for this complication. A postoperative CT scan could be a valuable asset to identify non-displaced fractures and maybe delay the rehabilitation protocol accordingly.

We do acknowledge limitations in our study. We operated on 15 hips, which is not enough to account for every possible complication. In addition, such a cadaveric model cannot identify non-macroscopic nerve damage (neuropraxia), since the diagnosis would depend on symptoms reported by the patient. It also cannot identify subtle vascular damage such as thrombosis due to intimal damage.

The average age of our patients was 65 years old. This is high when compared to the typical age of a patient that undergoes a PAO, which is approximately 30 years.^1^ Also, we suspect that some degree of osteoporosis among our specimens could be implicated on the occurrence of fractures. Unfortunately, we were not able to perform DEXA scans.

We were not able to obtain preoperative radiographs to evaluate the presence of signs of hip dysplasia. In specimens with normal or even excessive coverage, the margin of error for a PAO is even smaller due to the deepness of the acetabular socket. 

## CONCLUSIONS

The Bernese periacetabular osteotomy is a complex procedure associated with a learning curve, as shown by the reduction on complication rates after 15 procedures. There is a considerable risk of posterior column fracture and articular extension of the osteotomies. Doing the retroacetabular and ischial osteotomies from the posterior approach allows for improved visualization of the cuts, and this may be beneficial for surgeons in the beginning of their learning curve or for centers where this procedure is not done frequently. We recommend training this technique in multiple cadavers before performing it in live patients.
